# Psychotrauma update

**DOI:** 10.3402/ejpt.v6.27625

**Published:** 2015-04-08

**Authors:** Miranda Olff

On October 9, 2014, the second SCEM symposium on psychological traumatization was organized in Amsterdam, the Netherlands, chaired by Robbert-Jan Verkes, MD, PhD, and Anton van Balkom MD, PhD. The symposium was attended by over 100 participants: psychiatrists, medical doctors, psychologists, and psychiatric nurses.

This second symposium on the consequences of psychotrauma was organized because the first one focusing on the aspecific relationship between psychotrauma and psychopathology had been very successful. Apparently there is large interest in psychotrauma in the Netherlands (Olff & Vermetten, 2013), possibly explained by the trauma history of the country (Vermetten & Olff, 2013). At the first SCEM symposium, various subjects were presented ranging from the neurobiological sequelae of psychotrauma to evidence-based treatment of posttraumatic stress disorder (PTSD) and the findings of the government-installed research committee on sexual abuse in the Dutch Roman Catholic church.

In this second symposium, we focused on several topics relevant to both research and clinical practice. The first lecture addressed diagnostic issues of PTSD in recent history (Vermetten, 2015). The complex relationship between psychotrauma and psycho-active substance dependence was presented by Wim Van den Brink (2015). The question whether PTSD could be viewed as a memory disorder rather than an anxiety disorder was the topic of Hein Van Marle (2015). Miranda Olff presented research into the effect of intranasal oxytocin in recently traumatized as well as in PTSD patients (Olff et al., 2015). Ramón Lindauer (2015) went into the clinically interesting question of whether a child should receive imaginary exposure to psychotrauma for treatment as soon as possible or whether it would be better to stabilize the psychological situation first. Theo Ingenhoven (2015) commented on the treatment of PTSD-symptoms related to abuse and neglect in early youth within the psychotherapies for borderline personality disorder. Finally, Gert-Jan Hendriks, MD, PhD, presented data on the cognitive enhancement of exposure outcome with d-cycloserine in PTSD (Hendriks & de Kleine, 2015). The abstracts of these keynote lectures are presented in this issue.

*Miranda Olff* Editor-in-Chief
