# Corrigendum

**DOI:** 10.3402/ejpt.v6.28722

**Published:** 2015-06-19

Regarding the Editorial ‘Psychotrauma update’ by *Miranda Olff*

Published in *European Journal of Psychotraumatology* 2015. Citation: European Journal of Psychotraumatology 2015, **6**: 27625 - http://dx.doi.org/10.3402/ejpt.v6.27625

Not all authors were listed for this Editorial. The correct authors are displayed below.

Robbert-Jan Verkes, PhD

Department of Psychiatry Radboudumc

Law School Radboud University

Forensic Psychiatric Centre Pompestichting

Nijmegen, The Netherlands

Anton van Balkom, MD, PhD

Department of Psychiatry

VU-University Medical Centre and GGZ inGeest

Amsterdam, The Netherlands

Miranda Olff

Editor-in-Chief

Department of Psychiatry

Academic Medical Center, University of Amsterdam and

Arq Psychotrauma Expert Group

Diemen, The Netherlands

